# Longitudinal spin Seebeck effect contribution in transverse spin Seebeck effect experiments in Pt/YIG and Pt/NFO

**DOI:** 10.1038/ncomms9211

**Published:** 2015-09-23

**Authors:** Daniel Meier, Daniel Reinhardt, Michael van Straaten, Christoph Klewe, Matthias Althammer, Michael Schreier, Sebastian T. B. Goennenwein, Arunava Gupta, Maximilian Schmid, Christian H. Back, Jan-Michael Schmalhorst, Timo Kuschel, Günter Reiss

**Affiliations:** 1 Department of Physics, Center for Spinelectronic Materials and Devices, Bielefeld University, Universitätsstraße 25, 33615 Bielefeld, Germany; 2 Walther-Meissner-Institut, Bayerische Akademie der Wissenschaften, Walther-Meissner-Strasse 8, 85748 Garching, Germany; 3 Physik-Department, Technische Universität München, James-Franck-Str. 1, 85748 Garching, Germany; 4 Nanosystems Initiative Munich (NIM), Schellingstraße 4, 80799 München, Germany; 5 Center for Materials for Information Technology, University of Alabama, Tuscaloosa, Alabama 35487, USA; 6 Institute of Experimental and Applied Physics, University of Regensburg, Universitätsstraße 31, 93040 Regensburg, Germany

## Abstract

The spin Seebeck effect, the generation of a spin current by a temperature gradient, has attracted great attention, but the interplay over a millimetre range along a thin ferromagnetic film as well as unintended side effects which hinder an unambiguous detection have evoked controversial discussions. Here, we investigate the inverse spin Hall voltage of a 10 nm thin Pt strip deposited on the magnetic insulators Y_3_Fe_5_O_12_ and NiFe_2_O_4_ with a temperature gradient in the film plane. We show characteristics typical of the spin Seebeck effect, although we do not observe the most striking features of the transverse spin Seebeck effect. Instead, we attribute the observed voltages to the longitudinal spin Seebeck effect generated by a contact tip induced parasitic out-of-plane temperature gradient, which depends on material, diameter and temperature of the tip.

## Abstract (web summary)

Measurements of the spin Seebeck effect, the generation of spin current in a ferromagnet due to a temperature gradient, are often hindered by parasitic effects. Here, the authors use ferromagnetic insulators to show how transverse spin Seebeck measurements can be explained by the longitudinal spin Seebeck.

## Graphical abstract

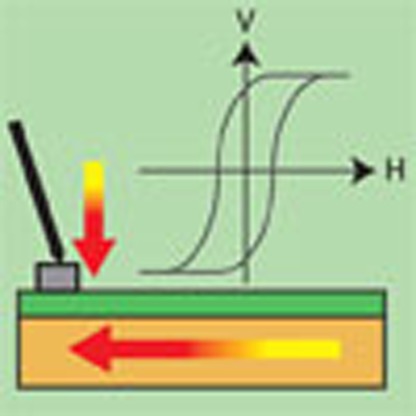

Spin caloritronics is an active branch in spintronics^1^,^2^. The interplay between heat, charge and spin transport opens a new area of fascinating issues involving the use of waste heat in electronic devices. One potentially useful effect for heat collecting^3^ is the spin Seebeck effect (SSE)^4^ which was observed in 2008. It was reported that a spin current perpendicular to an applied temperature gradient can be generated in a ferromagnetic metal (FMM) by the transverse SSE (TSSE)^4^. An adjacent normal metal (NM) converts the spin current via the inverse spin Hall effect (ISHE)^5^ into a transverse voltage *V*, which is antisymmetric with respect to the external magnetic field *H* (*V*(*H*)=−*V*(−*H*), see Fig. 1a). In this geometry, the temperature gradient is typically aligned in-plane (∇*T*_x_) and can also induce a planar Nernst effect (PNE) in FMM with magnetic anisotropy^6^ which is due to the anisotropic magnetic thermopower and symmetric with respect to *H* (*V*(*H*)=*V*(−*H*)). For pure ∇*T*_x_ in a ferromagnetic or ferrimagnetic insulator (FMI) there is no PNE, since there are no free charge carriers available. However, if the NM material is close to the Stoner criterion, a static magnetic proximity effect could induce a so called proximity PNE, which in general is present in spin polarized NM adjacent to a FMM and could also occur in a NM–FMI contact (Fig. 1a). Reports of previous experiments with pure ∇*T*_x_ can be found for NM/FMM^4^,^6^,^7^,^8^ and for NM/FMI^9^.

For the longitudinal SSE (LSSE)^10^ the spin current flows directly from the FM into an adjacent NM parallel to the temperature gradient (Fig. 1b), which is typically aligned out-of-plane (∇*T*_z_). In NM/FMM bilayers the anomalous Nernst effect (ANE) can occur, but is absent in the FMI. In semiconducting materials the ANE contributes to the LSSE as already shown for Pt/NiFe_2_O_4_ at room temperature^11^. In addition, if the NM would be spin polarized by the proximity to the FM, an additional proximity ANE could occur^12^ (Fig. 1b). Reports of previous experiments with pure ∇*T*_z_ can be found for NM/FMM^11^ and for NM/FMI^10^,^13^,^14^,^15^,^16^,^17^,^18^,^19^. As summarized in Fig. 1c an unintended ∇*T*_z_ can hamper the evaluation of TSSE experiments with applied ∇*T*_x_. Heat flow into the surrounding area or through the electrical contacts can induce an additional ANE in NM/FMM bilayers and NM/magnetic semiconductors as discussed in literature^8^,^20^,^21^,^22^,^23^,^24^. But since, in principle, all the effects of an LSSE experiment can be present in the TSSE experiment with unintended ∇*T*_z_, proximity Nernst effects and especially parasitic LSSE can also be present in NM/FMI bilayers as already mentioned recently^25^. This leads to four possible effects which are antisymmetric with respect to the external magnetic field, when the temperature gradient is not controlled very carefully (see Fig. 1c). The prevalence of multiple thermoelectric effects in conducting ferromagnets poses a significant challenge for identifying any individual contribution to the total voltage signal. Previous reports on this matter^6^,^8^,^21^,^22^,^23^ are thus limited in significance and could not unambiguously prove or disprove the existence of a TSSE.

Here we report on the investigation of the TSSE on the magnetic insulators Y_3_Fe_5_O_12_ (YIG) and NiFe_2_O_4_ (NFO). We observe the influence of out-of-plane temperature gradients when an in-plane temperature gradient is applied intentionally by varying the material, diameter and temperature of the electrical contact tips. We circumvent all of the above issues and are, for the first time, able to demonstrate that the LSSE alone accounts for the observed voltage signals. Thus, we fill a gap in the controversial discussion about TSSE investigations.

## Results

### Sample structure and characterization

**Figure 1:**
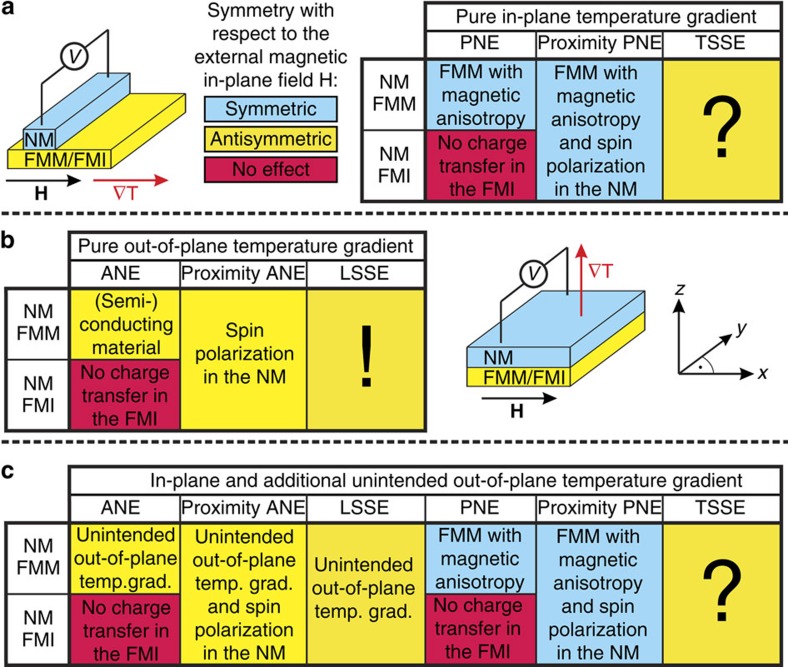
Summary of effects in SSE experiments. An overview of all possible effects is given for NM/FMM and NM/FMI bilayers depending on the symmetry of the transverse voltage *V* with respect to the external magnetic in-plane field *H* for antisymmetric magnetization reversal processes. A distinction is made between symmetric effects (blue), antisymmetric effects (yellow) and the effects, which are not possible in the considered system (red). (**a**) Pure in-plane ∇*T* along the *x* direction parallel to the magnetic field vector **H** for TSSE measurements. In NM/FMM systems a PNE due to magnetic anisotropy in the FMM or a proximity PNE due to the spin polarization in the NM can exist. In NM/FMI a PNE is absent due to the lack of charge carriers in the FMI. (**b**) Pure out-of-plane ∇*T* along the *z* direction perpendicular to the magnetic field vector **H** for LSSE measurements. In NM/FMM systems the ANE exists. In both NM/FMM and NM/FMI the proximity ANE can appear if the magnetic proximity effect generates a spin polarization at the interface. The LSSE is proved for NM/FMI systems and should be also present in NM/FMM systems. (**c**) In-plane and unintended out-of-plane ∇*T*. A combination of all effects from **a** and **b** has to be considered.

Magnetic insulators are of increasing importance for spintronics, magnonics and spin caloritronics^9^,^26^,^27^,^28^,^29^,^30^. The phenomena presented in Fig. 1 and the discussions of side effects in TSSE experiments have not been treated systematically in the literature for NM/FMI bilayers so far (Table 1). For the measurements in this work we used YIG films with a thickness of *t*_YIG_=180 nm. The films show a coercive field of about 100 Oe and a saturation magnetization of *M*_S_=120 kA m^−1^. As common for many thin films these values deviate slightly from bulk properties. Previous studies^14^,^15^ demonstrated, however, that the LSSE is very robust to variations in the magnetic properties. Our results should thus also be applicable to bulk samples and other thin films. YIG films with similar properties are also relevant for further spin transport phenomena, for example, the recently observed spin Hall magnetoresistance^31^,^32^. For reference measurements on another magnetic insulator system we used NFO films with a thickness of *t*_NFO_=1 μm and a coercive field of about 160 Oe. On top of the magnetic insulator systems a thin Pt strip with a thickness of *t*_Pt_=10 nm was deposited on one side as a spin detector material.

### Experimental setup

**Figure 2:**
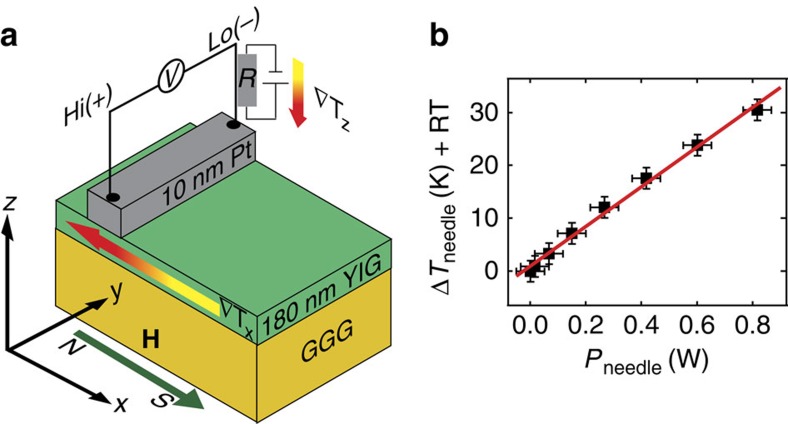
Measurement configuration. (**a**) In-plane temperature gradient ∇*T*_x_ applied parallel to the external magnetic field **H** in the *x* direction from the magnetic north (N) to the magnetic south (S) for positive magnetic fields **H** with respect to the measurement of the voltage *V* at the Pt strip between Hi (+) and Lo (−) of the multimeter. An additional out-of-plane temperature gradient ∇*T*_z_ can be induced by using thick contact tips or heating one tip with a voltage at a resistor *R* attached to the tip (only thermally connected, not electrically). (**b**) The temperature *T*_needle_ at the tip of the Au needle as a function of the power *P*_needle_ at the resistor *R* obtained in a reference measurement. The error bars mirror the systematical error of the temperature and power determination.

If not mentioned otherwise the experiments are performed under ambient conditions as described in (ref. 8) mirroring those of the very first TSSE measurements on FMIs by Uchida *et al.*^9^. The ends of the Pt strip (*l*_Pt_=5 mm) were contacted with a microprobe system with Au and W tips of different diameters. Furthermore, one Au tip was equipped with a 1.5-kΩ resistor for heating the tip (only thermally connected to the tip, not electrically) to intentionally induce a ∇*T*_z_ (Fig. 2a)^8^. Figure 2b shows the nearly linear relation between the power *P*_needle_ dissipated in the resistor and the tip temperature *T*_needle_ as determined with a type-K thermocouple glued to the tip in a calibration measurement. The heated Au needle is labelled *T*_needle_=RT+x with room temperature RT=296 K. The voltage *V* at the Pt strip was measured with a Keithley 2182A nanovoltmeter. An external magnetic field *H* in *x* direction was applied in a range of ±600 Oe for YIG and of ±1,000 Oe for NFO films. The voltage values *V*_sat_ quoted throughout the remainder of the manuscript are the respective averaged voltages levels at saturation.

### Measurements in vacuum

**Figure 3:**
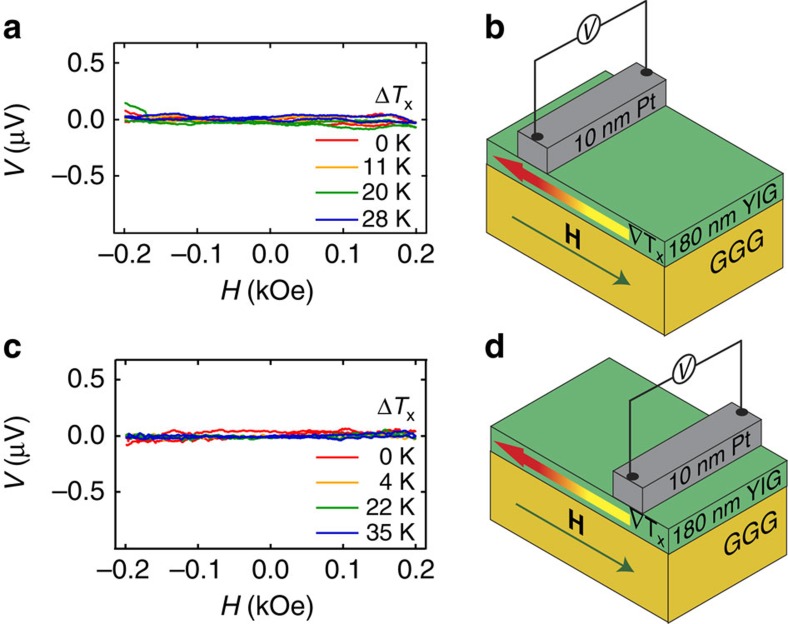
Pt/YIG contacted with Au bonding wires in vacuum. *H* dependence of *V* measured at the Pt strip on YIG using Au bonding wires with a diameter of 25 μm for various in-plane temperature differences Δ*T*_x_ performed under vacuum. (**a**) Measurements for Pt strip at the hot side of the YIG film. (**b**) Sample and measurement configuration for the data in **a** with the in-plane temperature gradient ∇*T*_*x*_ parallel to the external magnetic field **H**. (**c**) *H* dependence of *V* measured at the Pt strip located on the cold side of the YIG film for various in-plane temperature differences Δ*T*_x_. (**d**) Sample and measurement configuration for the data in **c**.

Before measurements under ambient conditions were performed we did reference measurements on Pt/YIG under vacuum. The Pt strip was contacted by Au bonding wires with 25 μm in diameter. The results are shown in Fig. 3 for Pt strip on the hot side (Fig. 3a) and Pt strip on the cold side of the YIG film (Fig. 3b) for various in-plane temperature gradients ∇*T*_*x*_. For both sample sides no significant effect within the measurement sensitivity limit below ±20 nV can be observed. The same behaviour was obtained for Pt/NFO where no significant effect could be observed for Pt strip on both sample ends (Supplementary Fig. 1).

### Measurements under ambient conditions

**Figure 4:**
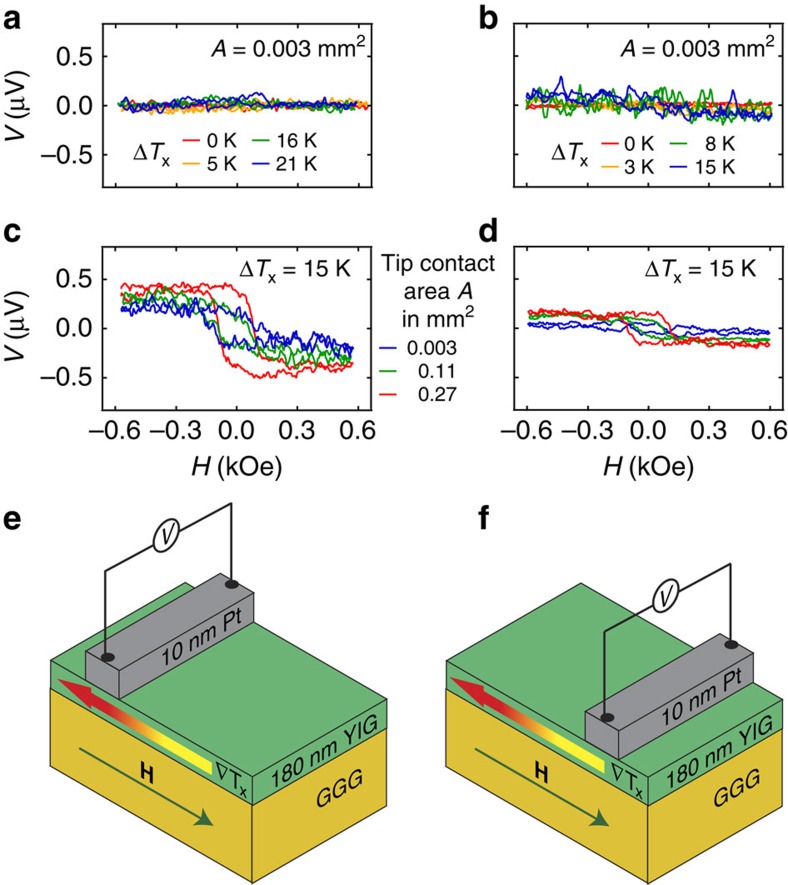
Pt/YIG contacted with W tips under ambient conditions. (**a**) *H* dependence of *V* measured at the Pt strip on YIG located at the hot side using thin W tips and low contact force (tip contact area of *A*=0.003 mm^2^) for various Δ*T*_x_. (**b**) Pt strip at the cold side and the same W tips used in **a** for various Δ*T*_x_ and using low contact force. (**c**) Pt strip at the hot side and different W tips with various contact areas with a fixed Δ*T*_*x*_=15 K using a high contact force. (**d**) Pt strip at the cold side with a fixed Δ*T*_*x*_=15 K and with the same W tips as in **c** using a high contact force. (**e**) Sample and measurement configuration for the data in **a** and **c** with the in-plane temperature gradient ∇*T*_*x*_ parallel to the external magnetic field **H**. (**f**) Sample and measurement configuration for the data in **b** and **d**.

Afterwards, the samples were mounted in the setup described in (ref. 8) performed under ambient conditions. First, the ISHE voltage from the Pt/YIG sample was measured for various Δ*T*_x_. The Pt strip was on the hot side and contacted with W tips carefully. Again, the voltage shows no significant variation within the sensitivity limit of about ±40 nV when *H* is varied (Fig. 4a). No Nernst effects are observed due to the insulating magnetic layer and no evidence for an additional ∇*T*_z_ can be detected. Therefore, the clamping and heating of the sample in our setup and the contacting with thin W tips results in a pure ∇*T*_x_ as already shown in (ref. 8). TSSE is not observable although the Pt strip is located on the hot side of the YIG film and far away from the center where the TSSE should vanish. The comparison with Pt strip at the cold sample end is shown in Fig. 4b for the thinnest W tips and different temperature gradients ∇*T*_*x*_. For the largest ∇*T*_*x*_ we can observe a noticeable effect given by a difference between the voltages in saturation for positive and negative magnetic fields.

### Contact area dependence

In the next step we contacted again and pushed the thin W tips with more pressure into the Pt strip, which increases the effective electrical contact area *A* (blue curve in Fig. 4c). Afterwards, the tips were changed by other W tips with a larger contact area. The observed voltages are plotted in Fig. 4c with Pt strip at the hot sample end and Δ*T*_*x*_=15 K. The voltages vary significantly when *H* is changed. *V* is antisymmetric with respect to *H* and the voltage in saturation increases for larger contact areas. In Fig. 4d the same increase of antisymmetric effect is obtained for different W tips with increasing contact area at the cold sample side, but the magnitude of effect is generally smaller compared with the hot side. However, the sign of *V* in saturation is the same for Pt strip at the hot and the cold side. This behaviour could be verified for Pt/NFO films (Supplementary Fig. 2) and excludes the TSSE which has to show a sign change in *V* between hot and cold side (Supplementary Note 1).

**Figure 5:**
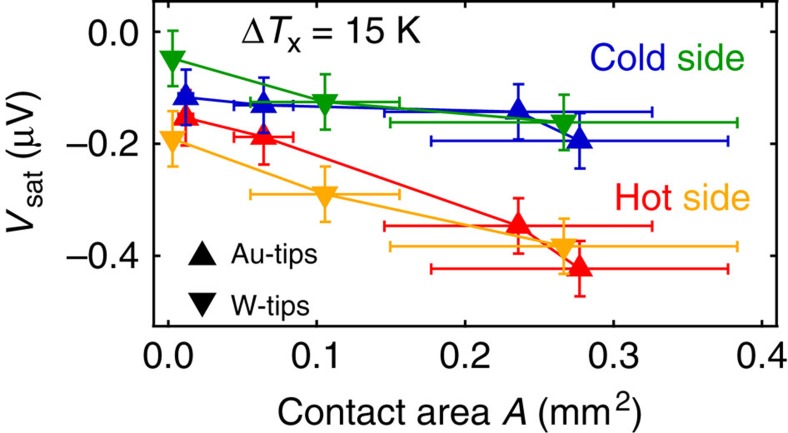
Influence of the contact area. *V*_sat_ as a function of the determined contact area *A* for various Au and W tips with different diameters for Pt strip at the hot and the cold side and a constant Δ*T*_x_=15 K in Pt/YIG. The deviation of the average contact area to the estimated areas is used as the experimental error which takes into account the difference of the contact area when the sample is recontacted or contacted with stronger pressure. The experimental error of *V*_sat_ was assumed to a value of ∼ ±50 nV which takes into account the estimated deviation between high and low contact force.

In Fig. 5 the magnitudes *V*_sat_ obtained for all W tips with different contact areas (Fig. 4) are plotted against the contact area *A* of the tip for Pt strip on the hot and cold side for a temperature difference Δ*T*_*x*_=15 K. Furthermore, a variation of Au tips with different contact areas were added which show the same behaviour of *V*_sat_ depending on *A* for both sample sides (Supplementary Fig. 3). Due to the fact that the real attached contact area could not 100% be reproduced we estimated the average between the possible maximum contact area and a sufficiently low area (Supplementary Fig. 4). This leads to relatively large error bars. However, for Pt strip on the hot and the cold side the sign of *V*_sat_ is the same. Furthermore, the absolute value of *V*_sat_ decreases for smaller contact areas of the used tips for both materials (W and Au). The effect size for Pt strip on the cold side is generally smaller compared with the hot side when the same temperature difference of Δ*T*_*x*_=15 K is applied. We explain this behaviour of *V*_sat_ by an unintended heat flux through the tips leading to a vertical temperature gradient ∇*T*_*z*_ and, therefore, to a LSSE induced spin current into the Pt.

Furthermore, in Fig. 5, *V*_sat_ does not tend to zero when *A* gets sufficiently small, which can be explained by an additional influence of ∇*T*_*z*_ contributions due to different thermal conductivities of the investigated film and substrate materials.

### Influence of contact tip heating

**Figure 6:**
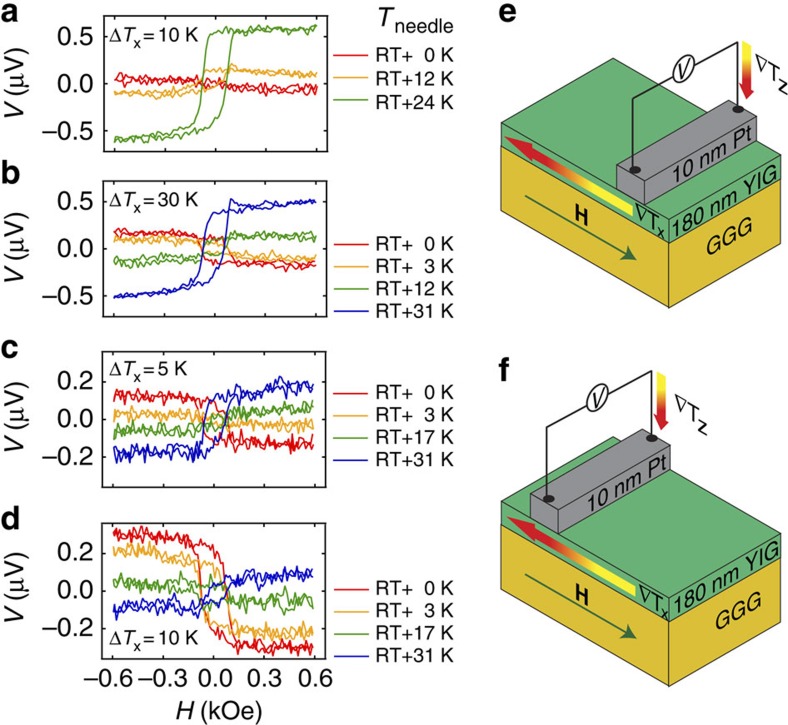
Pt/YIG contacted with heatable Au tips under ambient conditions. *H* dependence of *V* measured at the Pt strip on YIG using thick Au tips and different tip heating voltages inducing an additional ∇*T*_z_ for various ∇*T*_x_. (**a**) Pt strip at the cold side for Δ*T*_x_=10 K. (**b**) Pt strip at the cold side for Δ*T*_x_=30 K. (**c**) Pt strip at the hot side for Δ*T*_x_=5 K. (**d**) Pt strip at the hot side for Δ*T*_x_=10 K. (**e**) Sample and measurement configuration for the data in **a** and **b** with the in-plane temperature gradient ∇*T*_*x*_ parallel to the external magnetic field **H** and an additional out-of-plane temperature gradient ∇*T*_*z*_. (**f**) Sample and measurement configuration for the data in **c** and **d**.

In the next step we used the thickest Au tips (Fig. 6) with a resistor glued to the tip. The contact area *A* was about 0.28 mm^2^. It can be seen that the measured voltage is antisymmetric with respect to the magnetic field (Fig. 6a–d). Next, we applied a voltage to the Au tip resistor to increase the temperature of the tip and change the out-of-plane heat flow.

In Fig. 6a for *T*_needle_=RT a small antisymmetric effect of about *V*_sat_=−50 nV is obtained when the Pt strip is at the cold side of the YIG film. When the needle is heated to *T*_needle_=RT+12 K the ISHE voltage changes its sign to a value of *V*_sat_=+95 nV and changes further to *V*_sat_=+590 nV for *T*_needle_=RT+24 K. The Au needles with larger contact areas compared with thinner W tips or Au bonding wires generate an additional out-of-plane heat flow at the cold side of the sample. This heat flow changes its sign with increasing *T*_needle_, which can be detected by the sign reversal of the measured voltage. When ∇*T*_x_ is increased (Δ*T*_x_=30 K in Fig. 6b) the ISHE voltage at the Pt is *V*_sat_=−170 nV for *T*_needle_=RT and therefore three times larger than for Δ*T*_x_=10 K. The ISHE voltage again increases with increasing *T*_needle_ and changes sign.

For a Pt strip at the hot side *V*_sat_ without tip heating is larger than at the cold side. For Δ*T*_x_=5 K the magnitude is about *V*_sat_=−130 nV (Fig. 6c) and can be decreased to *V*_sat_=−300 nV for Δ*T*_x_=10 K (Fig. 6d). The sign and the magnitude of *V*_sat_ can also be controlled by *T*_needle_ and, therefore, by ∇*T*_z_. When *T*_needle_ is fixed at RT+31 K, *V*_sat_ is about +180 nV for Δ*T*_x_=5 K and +90 nV for Δ*T*_x_=10 K.

**Figure 7:**
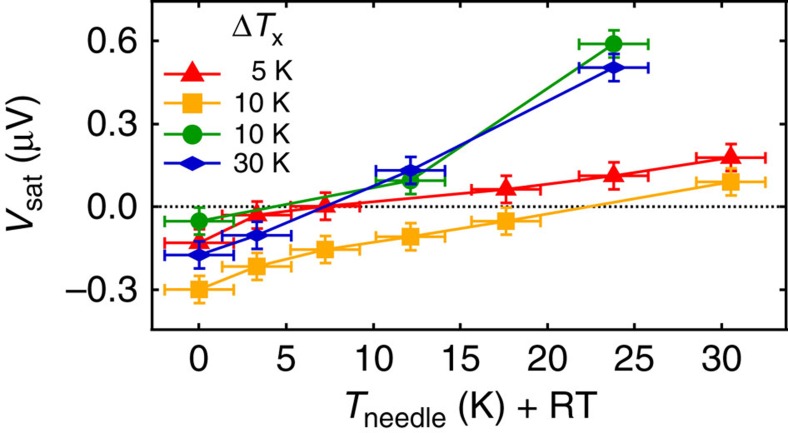
Influence of contact tip heating. *V*_sat_ as a function of the Au needle temperature *T*_needle_ for various in-plane temperature differences Δ*T*_*x*_ in Pt/YIG and Pt strip on the hot (red triangles, yellow squares) and on the cold sample side (green circles, blue rhombuses). The experimental error of *T*_needle_ was estimated in the reference measurement and describes the deviation from the average value over several measurements. The experimental error of *V*_sat_ describes the deviation between high and low contact forces.

*V*_sat_ measured for Pt/YIG at the hot and cold side is plotted as a function of *T*_needle_ for different Δ*T*_x_ in Fig. 7. A non-heated Au needle results in the same sign of *V*_sat_ for all Δ*T*_x_, while |*V*_sat_| is smaller on the cold side compared with the hot side. Again, this behaviour can be explained by an unintended heat flux through the Au needles creating a vertical temperature gradient ∇*T*_z_ and thereby an LSSE voltage. We note that this interpretation also holds when a rigorous sign check is applied^33^.

For a heated Au needle *V*_sat_ increases and crosses zero (Fig. 7). Here the tip heating compensates the out-of-plane heat flux induced by Δ*T*_x_ (∇*T*_z_=0). After the sign change of *V*_sat_ (and therefore, ∇*T*_z_), the values increase with a larger (smaller) slope for the cold (hot) side. The temperature difference between the sample at the hot side and the non-heated Au tip, which is at room temperature, is larger compared with the temperature difference between the colder sample side, which is closer to room temperature, and the non-heated Au tip, which is at room temperature. When the Au tip is heated on the hot sample side the amount of heating power is larger to reverse the direction of the out-of-plane temperature gradient than on the cold sample side. Therefore, the slope of the curves in Fig. 7 for Au tip at the cold sample side is larger compared with the slope of the curves for Au tip at the hot sample side.

### Calculation of the magnon-electron temperature difference

Xiao *et al.*^34^ discussed the temperature difference Δ*T*_me_ between the magnon temperature in the FM and the electron temperature in the NM as the origin of the thermally induced spin current. Δ*T*_me_ can be inferred from the recorded voltage as^25^,^34^


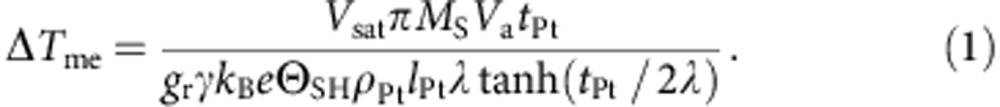


Here *V*_a_ is the magnetic coherence volume, *g*_r_ is the real part of the spin mixing conductance, γ is the gyromagnetic ratio, *k*_B_ is the Boltzmann constant, *e* is the elementary charge, Θ_SH_ is the spin Hall angle, *ρ*_Pt_ is the resistivity of the sample and λ is the spin diffusion length of the NM material. The two temperature model has proven successfully in relating Δ*T*_me_ to the phonon temperature, accessible in experiments^25^,^35^. We simulate the phonon and magnon temperatures assuming one-dimensional transport in our films and disregard the influence of thermal contact resistance other than the coupling between magnons and electrons. This yields a value Δ*T*_z_, the (phonon) temperature drop across the YIG film, for which the experimentally measured *V*_sat_ is obtained due to the LSSE. We assume *ρ*_Pt_=40 μΩm. All material dependent YIG parameters were taken from (ref. 25).

We calculated Δ*T*_me_ for the largest and smallest *V*_sat_ taken from Fig. 7 at RT (red curves in Fig. 6a,d) and the corresponding Δ*T*_z_. For |*V*_sat_|=50 nV (Fig. 6a) we obtain Δ*T*_me_=0.5 μK and a corresponding Δ*T*_z_=2 mK. For |*V*_sat_|=300 nV (Fig. 6d) the corresponding values are Δ*T*_me_=3.2 μK and Δ*T*_z_=12 mK. The obtained Δ*T*_z_ are in the order of a few millikelvins. It is reasonable to assume that such values can be induced by for example, thick contact tips, especially considering that our initial simplifications should lead to an overestimation of Δ*T*_z_^25^. These calculations for the TSSE configuration were investigated in detail in (ref. 25). It was found that for Δ*T*_x_=20 K the obtained Δ*T*_me_ is well below 1 μK, even at the very edge of the sample where Δ*T*_me_ is maximized. This further supports the notion that spurious out-of-plane temperature gradients are responsible for the voltages observed in our samples.

### Measurements on Pt/NFO

For Pt/NFO a similar behaviour of *V*_sat_ could be observed for various combinations of in-plane temperature gradient, Au needle temperature and Pt strip location (hot or cold side) (Supplementary Fig. 5, Supplementary Note 2). The magnitudes of *V*_sat_ obtained for Pt/NFO are about an order of magnitude larger when compared with *V*_sat_ in Pt/YIG (see Supplementary Fig. 2 and Supplementary Fig. 5) for similar values Δ*T*_*x*_ and *T*_needle_. This difference can be explained by different film thicknesses and thermal conductivities of NFO and YIG as well as by different spin mixing conductances^36^,^37^.

## Discussion

For all experiments we performed in vacuum and using thin bonding wires for the electrical contacts, we could not observe any evidence for a TSSE as well as any other transport phenomena shown in Fig. 1. For the most experiments we performed under ambient conditions and using contact tips, we can clearly observe an antisymmetric behaviour of the voltage *V* with respect to the external magnetic field *H* which can be addressed to the LSSE due to an unintended out-of-plane temperature gradient Δ*T*_z_. For all remaining experiments, there is no evidence for any transport phenomena given in Fig. 1. For both investigated sample systems the spin mixing conductance is large enough to observe a thermally driven spin current across the NM/FMI interface as proven by the results of experiments with LSSE configuration for Pt/YIG (Supplementary Fig. 6) and Pt/NFO^11^, respectively.

In addition to the LSSE, we now discuss other parasitic effects like the ANE and proximity ANE, which can be produced by an unintended Δ*T*_z_ (see Fig. 1). We can exclude an ANE for YIG due to the lack of charge carriers. For NFO we observed an ANE which is one order of magnitude smaller at RT than the LSSE^11^. This ANE can be explained by the weak conductance of NFO at RT due to thermal activation energies of a few hundred meV depending on the preparation technique^11^,^29^. Absence of proximity ANE in Pt/NFO is ensured by X-ray resonant magnetic reflectivity measurements finding no interface spin polarization with the experimental limit of 0.02*μ*_B_ per Pt atom^38^. In case of Pt/YIG Geprägs *et al.* presented X-ray magnetic circular dichroism measurements (XMCD) with no evidence for any spin polarization in Pt^39^, while Lu *et al.* could show XMCD measurements indicating magnetic moments in Pt on their YIG samples^40^. Future investigations with X-ray resonant magnetic reflectivity can give more insight to this discrepancy. However, Kikkawa *et al.*^41^could show that a potential contribution of a proximity ANE additional to the LSSE is negligibly small. This supports our conclusion that the main antisymmetric contribution in our measurements on both Pt/YIG and Pt/NFO is the LSSE, which is driven by an out-of-plane temperature gradient.

We do not observe any symmetric contribution for ∇*T*_x_ without tip heating. Therefore, PNE and proximity PNE contributions can also be excluded. Nevertheless, we find a small symmetric contribution for strong tip heating as demonstrated in Fig. 6b for Δ*T*_needle_=RT+31 K. In the region of *H*_*C*_ small peaks are visible under symmetrization of the voltage. This hints at the existence of an additional magnetothermopower effect potentially induced by a temperature gradient ∇*T*_y_ along the Pt strip and will be part of future investigations.

Recently, Wegrowe *et al.*^42^ used anisotropic heat transport as an interpretation for the measured voltages using in-plane temperature gradients. In their work, they derived the anisotropic field-dependent temperature gradient in FMM and FMI from the Onsager reciprocity relations. Therefore, the thermocouple effect between the FM, the NM and the contacting tips can generate field-dependent voltages if there is a difference in the Seebeck coefficients. In our investigated systems, the Seebeck coefficients are indeed different for FM, Pt and the contact tips. However, since we do not observe a significant field-dependent variation of the ISHE voltage when the samples are bonded or carefully contacted with W tips, any anisotropic field-dependent heat-transport can be excluded as the reason for the observed voltages.

In summary, we investigated the relevance of TSSE in Pt/YIG and Pt/NFO systems. We found no significant ISHE voltages upon applying an in-plane temperature gradient and using 25-μm thin Au bonding wires or sharp W tips (0.003-mm^2^ contact area) as electric contacts. Increasing the contact area (up to 0.28 mm^2^), however, induces an additional out-of-plane heat flux accompanied by a LSSE ISHE voltage. This antisymmetric effect can be identified as LSSE which was verified by controlling the needle temperature, or changing the tip diameter accompanied by the contact area and therefore varying the out-of-plane temperature gradient. Taken together, in all our experiments, we thus only observe LSSE-type signatures. These LSSE voltages can be reminiscent of a TSSE-type response if an unintentional (or intentional) ∇*T*_z_ is present. This shows that utmost care is required if one is to interpret magnetothermopower effects in terms of the TSSE.

## Methods

### Sample fabrication

The YIG films were deposited on gadolinium gallium garnet (Gd_3_Ga_5_O_12_) (111)-oriented single crystal substrates with width and length *w*=*l*=5 mm by pulsed laser deposition from a stoichiometric polycrystalline target. The NFO films with a thickness of about *t*_NFO_=1 μm were deposited on 10 × 5 mm^2^ MgAl_2_O_4_ (100)-oriented substrates by direct liquid injection-chemical vapour deposition (DLI-CVD)^11^,^43^. After a vacuum break and cleaning with ethanol in an ultrasonic bath a *t*_Pt_=10 nm thin Pt strip was deposited by dc magnetron sputtering in an Ar atmosphere of 1.5 × 10^−3^ mbar through a 100 μm wide split-mask on one sample side of the YIG and NFO films with a length of *l*_Pt_=5 mm.

## Additional information

**How to cite this article:** Meier, D. *et al.* Longitudinal spin Seebeck effect contribution in transverse spin Seebeck effect experiments in Pt/YIG and Pt/NFO. *Nat. Commun.* 6:8211 doi: 10.1038/ncomms9211 (2015).

## Supplementary Material

Supplementary Information Supplementary Figures 1-6 and Supplementary Notes 1-2

## Figures and Tables

**Table 1:** Publications on TSSE experiments in different materials.

	**First report of TSSE**	**No TSSE found**
Metal	Uchida K. *et al.*^4^	Huang S. *et al.*^21^
		Avery A. *et al.*^6^
		Schmid M. *et al.*^22^
		Meier D. *et al.*^8^
		Bui C.T. *et al.*^23^
Semiconductor	Jaworski C.M. *et al.*^7^	Soldatov I.V. *et al.*^24^
Insulator	Uchida K. *et al.*^9^	Meier D. *et al.* (this work)

TSSE, transverse spin Seebeck effect.

The first reports of the TSSE are listed for the material classes metal, semiconductor and insulator. These publications are opposed to reports with the attempt to reproduce their observations investigated on equal material systems or similar material classes. These opposing publications have reported that there was no evidence of a TSSE in their measurements.
