# Supplementary material for: Longitudinal spin Seebeck effect contribution in transverse spin Seebeck effect experiments in Pt/YIG and Pt/NFO
Source: Nat Commun. 2015 Sep 23;6:8211. doi: 10.1038/ncomms9211 (PMC4598359; doi:10.1038/ncomms9211)
Supplement: Supplementary Information — Supplementary Figures 1-6 and Supplementary Notes 1-2 [file ncomms9211-s1.pdf]

Supplementary Figures

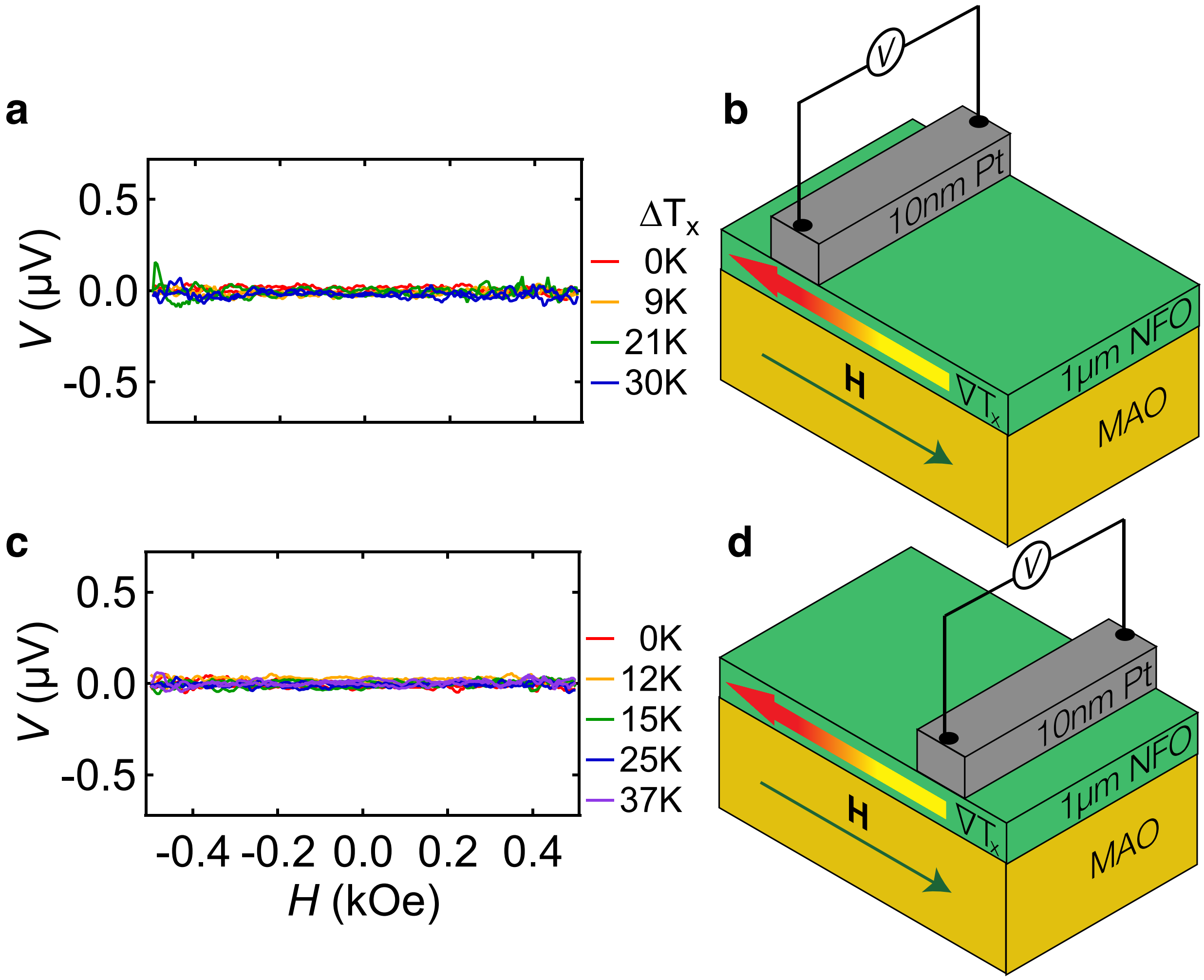

Supplementary Figure 1. Pt/NFO contacted with Au bonding wires in vacuum. *V* as a function of the external magnetic field *H* measured at the Pt strip on NFO with various temperature differences ΔT_x performed in vacuum. **(a)** Pt strip on the hot sample side. **(b)** Sample and measurement configuration for the data in **a** with the in-plane temperature gradient □T_x parallel to the external magnetic field **H**. **(c)** Pt strip on the cold side. **(d)** Sample and measurement configuration for the data in **c**. The Pt strip was contacted by thin Au bonding wires with a diameter of 25 µm. There is no significant effect observed within the measurement sensitivity limit of about ±20 nV.

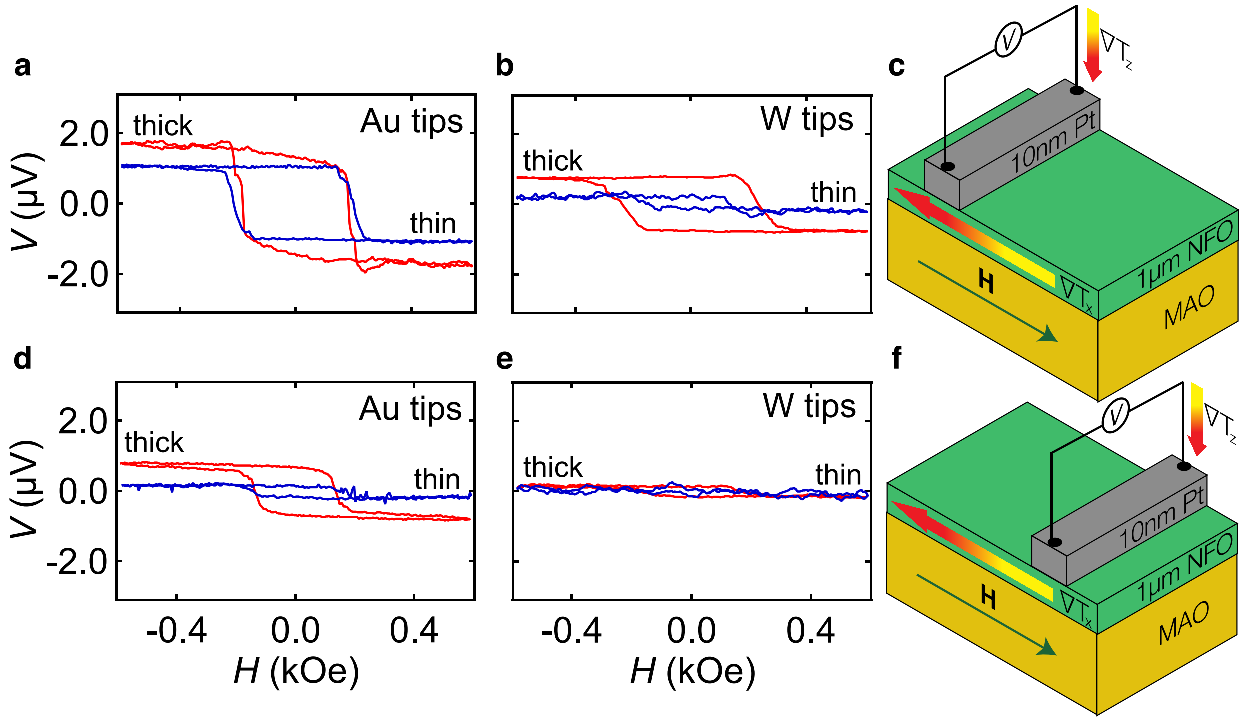

Supplementary Figure 2. Contact area dependence for Au and W tips on Pt/NFO. *V* as a function of the external magnetic field *H* measured at the Pt strip on NFO performed under ambient conditions for ΔT_x = 15 K. **(a)** The Pt strip was located on the hot sample side and contacted with thick (red) and thin (blue) Au tips which results in different contact areas of A = 0.28 mm^2 and A = 0.01 mm^2, respectively. **(b)** The Pt strip was also located on the hot sample side but contacted with thick (red) and thin (blue) W tips with contact areas of A = 0.11 mm^2 and A = 0.003 mm^2, respectively. **(c)** Sample and measurement configuration for the data in **a** and **b** with a fixed in-plane temperature gradient □T_x parallel to the external magnetic field **H.** **(d)** Pt strip located on the cold sample side contacted with thick and thin Au tips. **(e)** Pt strip located on the cold sample side contacted with thick and thin W tips. **(f)** Sample and measurement configuration for the data in **d** and **e**.

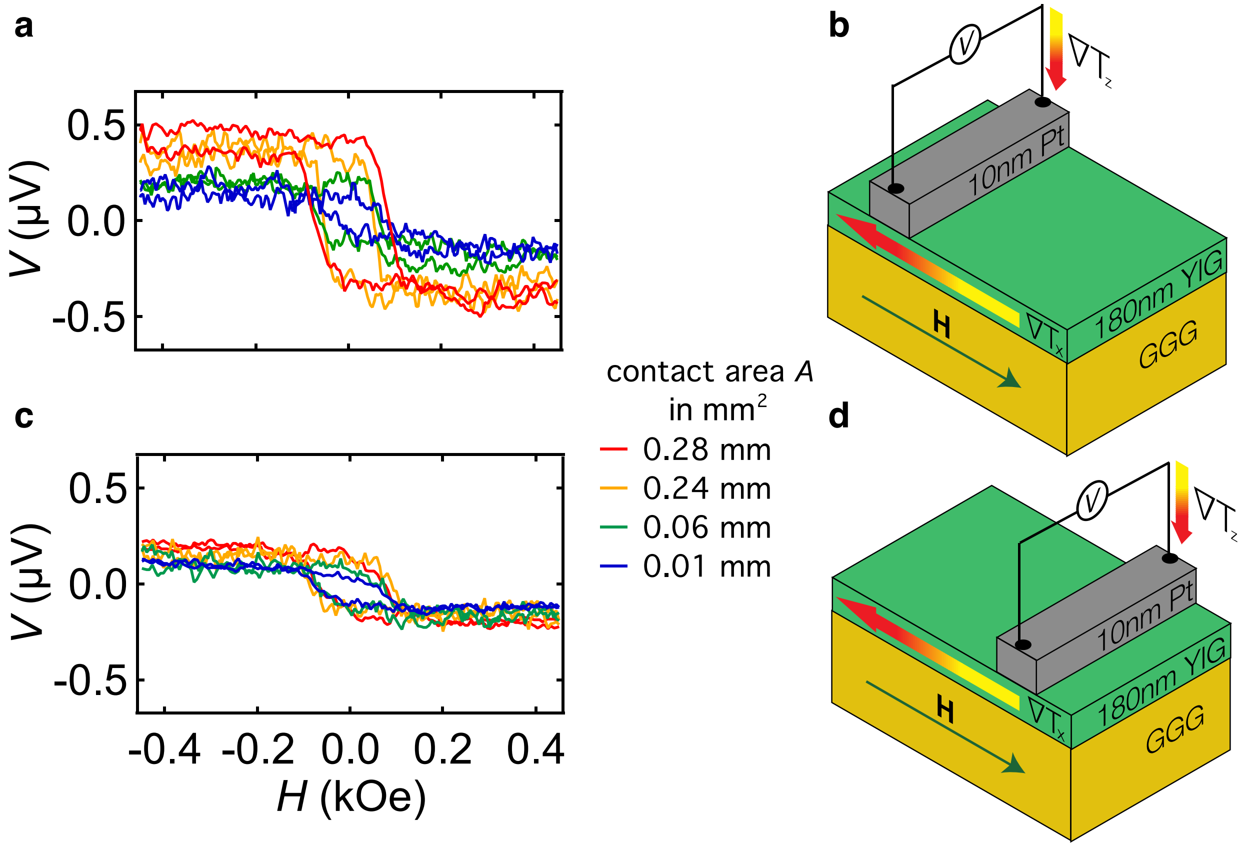

Supplementary Figure 3. Contact area dependence of Au tips on Pt/YIG. Voltage *V* measured as a function of the external magnetic field *H* with a constant in-plane temperature gradient ΔT_x = 15 K and Pt strip on the hot and the cold sample side. The Pt strip was contacted by Au tips with different diameters and therefore, different contact areas *A*. **(a)** The Pt strip is located on the hot sample side. The voltage *V* in saturation gets larger for increasing contact areas of the Au tips. **(b)** Sample and measurement configuration for the data in **a** with a fixed in-plane temperature gradient □T_x parallel to the external magnetic field **H**. **(c)** The Pt strip is located on the cold sample side. The voltage *V* in saturation increases with larger contact areas of the Au tips but the effect is smaller compared to Pt strip on the hot sample side. This is a result of the smaller temperature difference between sample and Au tips which results in a smaller out-of-plane temperature gradient □T_z. **(d)** Sample and measurement configuration for the data in **c**.

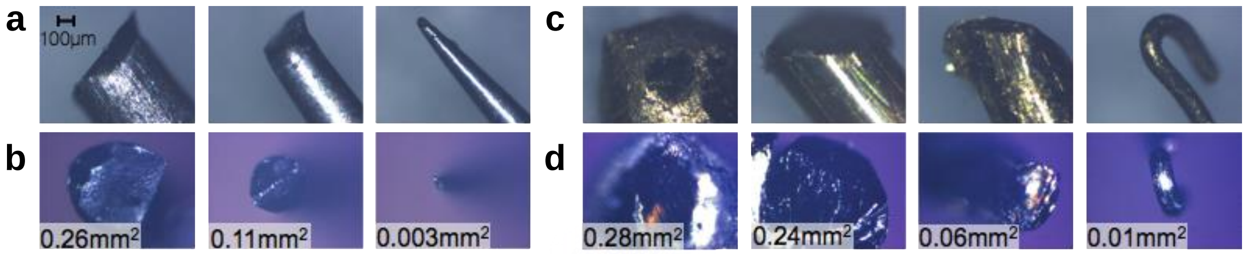

Supplementary Figure 4. Au and W tip diameters. (a) W tips with different contact areas *A* from the side view. **(b)** W tips from a view on top of the contact are. **(c)** Au tips with different diameters and different contact areas *A* from the side view. **(d)** Au tips from a view on top of the contact area. We calculated an average area of the maximum area of the complete observed tip and a minimal area of one edge of the tips. The deviation of the average value to the estimated areas is used as the experimental error which takes into account the difference of the contact area when the sample is recontacted or contacted with stronger pressure.

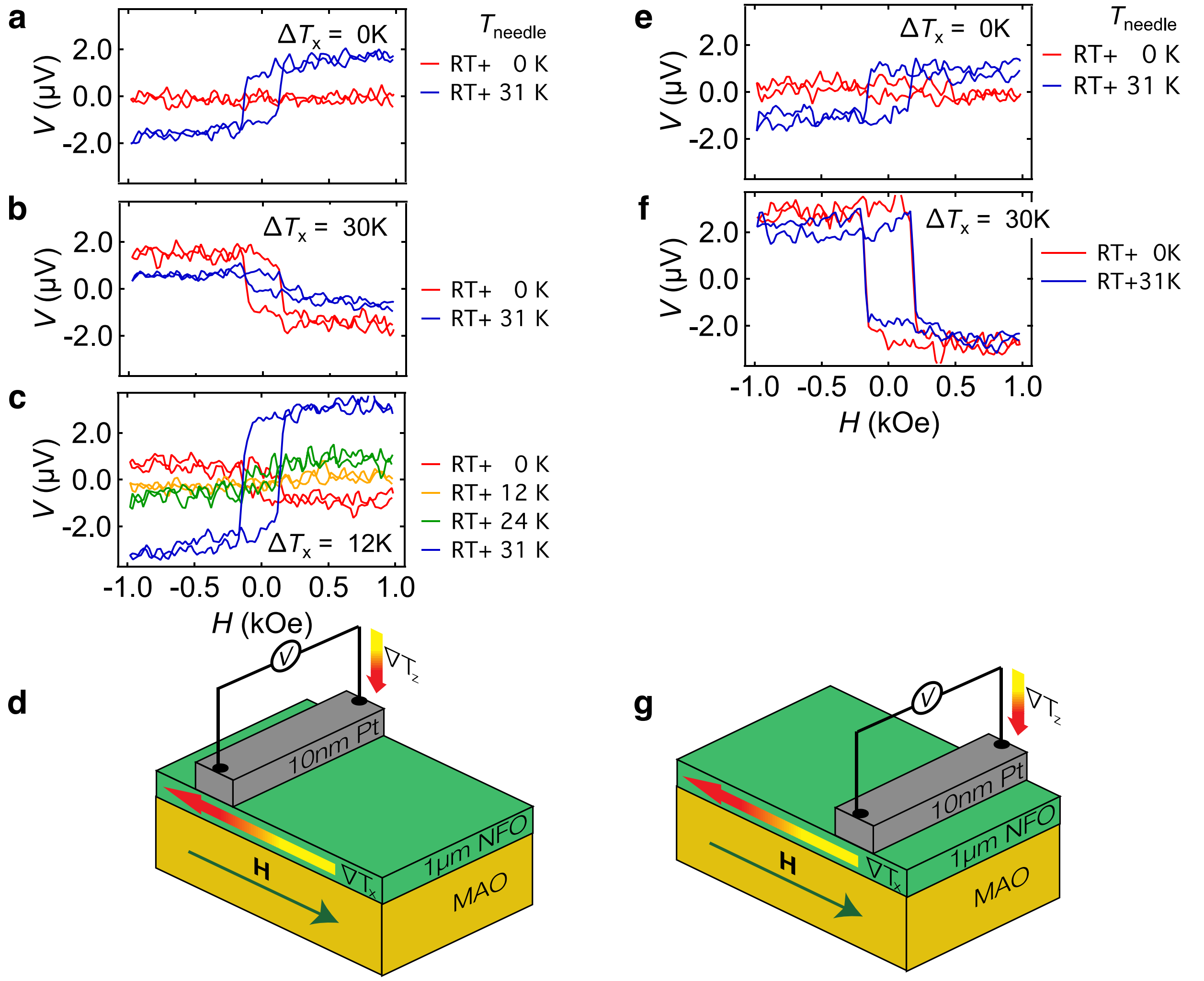

Supplementary Figure 5. Au tip heating dependence on Pt/NFO. (a) Voltage V as a function of the external magnetic field H measured under ambient conditions with heatable Au tips with a contact area of A = 0.28 mm^2 without an in-plane temperature gradient (ΔT_x = 0 K) and Pt strip on the hot sample side. One measurement was obtained without tip heating (T_{needle} = RT + 0 K) and shows no observable effect (red curve). The other measurement shows an antisymmetric behaviour in V with respect to H (blue curve). **(b)** The Pt strip was located on the hot sample side with an in-plane temperature difference of ΔT_x = 30 K for a needle temperature of T_{needle} = RT + 0 K (red curve) and T_{needle} = RT + 31 K (blue curve). **(c)** Measurements for various Au tip temperatures T_{needle} but a constant in-plane temperature difference ΔT_x = 12 K and Pt strip located on the hot side. **(d)** Sample and measurement configuration for the measurements in **a**, **b** and **c** with a fixed in-plane temperature gradient □T_x parallel to the external magnetic field **H** and an additional out-of-plane temperature gradient □T_z. **(e)** Measurements without an in-plane temperature difference (ΔT_x = 0 K), with and without Au tip heating (T_{needle} = RT + 0 K and T_{needle} = 31 K) and Pt strip located on the cold sample side. **(f)** Measurements with an in-plane temperature difference of ΔT_x = 30 K, with and without Au tip heating (T_{needle} = RT + 0 K and T_{needle} = 31 K) and Pt strip located on the cold sample side. **(g)** Sample and measurement configuration for the data in **e** and **f** with Pt strip located on the cold sample side.

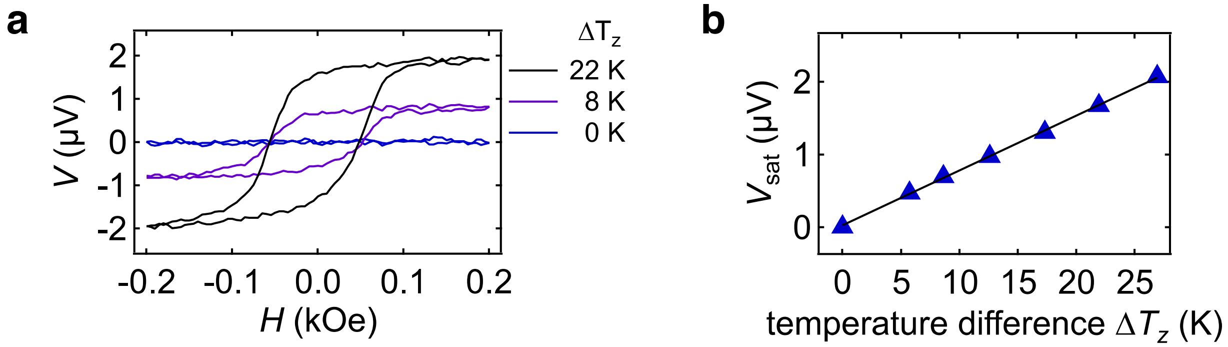

Supplementary Figure 6. Direct LSSE measurements on Pt/YIG. The investigated Pt/YIG sample was further measured in the LSSE configuration. Therefore, the sample was clamped between two copper blocks. The Pt strip was electrically isolated from the top copper block by a sapphire substrate. The temperature difference ΔT_z was measured between the copper blocks by two K-type thermocouples. The Pt strip was contacted by thin Au bonding wires at the free ends which were not covered by the top copper block. An external magnetic field H was applied in the film plane perpendicular to the Pt strip. Therefore, the direction of the temperature gradient, external magnetic field and electrical contacts fulfil the criteria for the ISHE to measure the LSSE. **(a)** LSSE measurements for three different in-plane temperature gradients are shown when the top copper block is heated. The typical LSSE behaviour shows a switching at H_C and a saturation at large magnetic fields. **(b)** The voltage V_{sat} in saturation is proportional to the applied temperature difference ΔT_z. The sign of the voltage V corresponds to previous measurements in TSSE configuration when an out-of-plane temperature gradient □T_z is involved which generates an LSSE. The same sign in V was obtained for heated Au tips when the tips were hotter than the sample. This results in the same direction for the out-of-plane temperature gradient.

Supplementary Notes

Supplementary Note 1. Contact area dependence on Pt/NFO

The NFO film was measured under ambient conditions. The Pt strip which was located on the hot (Supplementary Fig. 2 a, b) and on the cold sample side (Supplementary Fig. 2 d, e) was contacted with thick and thin Au and W tips, respectively. A fixed in-plane temperature difference of about ΔT_x = 15 K was applied. It can be observed that the antisymmetric effect of V with respect to H decreases for thinner tips and therefore with smaller contact areas. Furthermore, the antisymmetric effect decreases when the Pt strip moved from the hot to the cold side. The temperature difference between the sample and the contact tip gets smaller from the hot to the cold side which results in a smaller out-of-plane temperature gradient and a decreasing effect. The out-of-plane heat flow increases with a larger contact area which results in a larger effect. The contact area for the Au tips are A = 0.28 mm^2 and A = 0.01 mm^2 and for the W tips A = 0.11 mm^2 and A = 0.003 mm^2 for the thicker and the thinner tip, respectively. We want to emphasize that the sign of V does not change when the Pt strip moves from the hot to the cold side. This is the most striking behaviour of the TSSE which, therefore, can be excluded.

Supplementary Note 2. Au tip heating dependence on Pt/NFO

Further measurements on NFO were done with a heatable Au tip with a contact area of about A = 0.28 mm^2. In Supplementary Fig. 5 (a) the Pt strip was located on the hot side. No in-plane temperature gradient was applied. Without a heated Au tip no significant effect can be observed. However, when the Au tip is heated an antisymmetric effect in V with respect to H is obtained which has the opposite sign compared to measurements in Supplementary Fig. 2. Here, the out-of-plane temperature gradient is reversed due to the hotter tip compared to the non-heated sample. The same behaviour can be observed when the Pt strip is located on the cold sample side (Supplementary Fig. (e)). When an in-plane temperature gradient is applied the same sign of antisymmetric effect can be obtained which was observed with different Au and W tips in Supplementary Fig. 2 (see also Supplementary Fig. 5 (b) and 5 (f)). The out-of-plane temperature difference between the sample and the tip which is caused by the hotter sample is reduced by heating the Au tip. This reduction of ΔT_z results in a decreasing of the antisymmetric effect. A sign reversal in V can be obtained for a lower in-plane temperature difference of about ΔT_x = 12 K and an increasing needle temperature (Supplementary Fig. 5 (c)). The sign of V mirrors the direction of the out-of-plane heat flow given by the temperature difference between the sample and the tips.
